# Protein carbonylation causes sarcoplasmic reticulum Ca2+ overload by increasing intracellular Na+ level in ventricular myocytes

**DOI:** 10.21203/rs.3.rs-3991887/v1

**Published:** 2024-03-01

**Authors:** Elisa Bovo, Jaroslava Seflova, Seth L. Robia, Aleksey V. Zima

**Affiliations:** Loyola University Chicago, Stritch School of Medicine; Loyola University Chicago, Stritch School of Medicine; Loyola University Chicago, Stritch School of Medicine; Loyola University Chicago, Stritch School of Medicine

**Keywords:** heart, Ca2+ signaling, Na+ homeostasis, Na+/Ca2+ exchanger, Na+ pump, sarcoplasmic reticulum, methylglyoxal

## Abstract

Diabetes is commonly associated with an elevated level of reactive carbonyl species due to alteration of glucose and fatty acid metabolism. These metabolic changes cause an abnormality in cardiac Ca^2+^ regulation that can lead to cardiomyopathies. In this study, we explored how the reactive α-dicarbonyl methylglyoxal (MGO) affects Ca^2+^ regulation in mouse ventricular myocytes. Analysis of intracellular Ca^2+^ dynamics revealed that MGO (200 μM) increases action potential (AP)-induced Ca^2+^ transients and sarcoplasmic reticulum (SR) Ca^2+^ load, with a limited effect on L-type Ca^2+^ channel-mediated Ca^2+^ transients and SERCA-mediated Ca^2+^ uptake. At the same time, MGO significantly slowed down cytosolic Ca^2+^ extrusion by Na^+^/Ca^2+^ exchanger (NCX). MGO also increased the frequency of Ca^2+^ waves during rest and these Ca^2+^ release events were abolished by an external solution with zero [Na^+^] and [Ca^2+^]. Adrenergic receptor activation with isoproterenol (10 nM) increased Ca^2+^ transients and SR Ca^2+^ load, but it also triggered spontaneous Ca^2+^ waves in 27% of studied cells. Pretreatment of myocytes with MGO increased the fraction of cells with Ca^2+^ waves during adrenergic receptor stimulation by 163%. Measurements of intracellular [Na^+^] revealed that MGO increases cytosolic [Na^+^] by 57% from the maximal effect produced by the Na^+^-K^+^ ATPase inhibitor ouabain (20 μM). This increase in cytosolic [Na^+^] was a result of activation of a tetrodotoxin-sensitive Na^+^ influx, but not an inhibition of Na^+^-K^+^ ATPase. An increase in cytosolic [Na^+^] after treating cells with ouabain produced similar effects on Ca^2+^ regulation as MGO. These results suggest that protein carbonylation can affect cardiac Ca^2+^ regulation by increasing cytosolic [Na^+^] via a tetrodotoxin-sensitive pathway. This, in turn, reduces Ca^2+^ extrusion by NCX, causing SR Ca^2+^ overload and spontaneous Ca^2+^ waves.

## INTRODUCTION

Regular heart contraction critically depends on well-controlled intracellular Ca^2+^ ([Ca^2+^]_i_) homeostasis. In adult ventricular myocytes, the majority of Ca^2+^ that triggers contraction during systole is released from the sarcoplasmic reticulum (SR) through the ryanodine receptor (RyR2) Ca^2+^ release channel. It occurs as a result of RyR2 activation by Ca^2+^ influx through the voltage-gated L-type Ca^2+^ channels (LTCC) during the action potential [6]. During diastole, the excessive cytosolic Ca^2+^ is removed by the sarco/endoplasmic reticulum Ca^2+^-ATPase (SERCA2a) and the Na^+^/Ca^2+^ exchanger (NCX) [6], so heart can relax and fill with blood. It is well established that defects in [Ca^2+^]_i_ regulation can lead to arrythmias and contractile dysfunctions in many cardiac pathologies [1, 2, 5, 7, 8, 12, 37]. In cardiomyocytes, several important sarcolemmal transport mechanisms, including NCX, are coupled with the inward Na^+^ gradient. The gradient is established and maintained by the Na^+^/K^+^ ATPase (NKA), also called Na^+^ pump [32]. As a result, an inhibition of the Na^+^ pump causes accumulation of intracellular Na^+^ ([Na^+^]_i_) and, therefore, reduction of Ca^2+^ extrusion by NCX. Selective and moderate inhibition of NKA with glycosides has been traditionally used as a strategy to increase [Ca^2+^]_i_ and heart contraction in patients with chronic heart failure (HF) [18]. On the another hand, excessive [Na^+^]_i_ accumulation during myocardial infarction plays a critical role in cytosolic Ca^2+^ overload and myocardial injury [21]. Furthermore, several models of HF are associated with abnormal Na^+^ regulation and increased [Na^+^]_i_ [13, 22]. Thus, well-controlled intracellular Ca^2+^ and Na^+^ homeostasis are critically important for proper heart function.

More than 500 million people around the world live with type 1 and type II diabetes, the chronic conditions that affect the ability of the body to produce or sense insulin and, therefore, control of the blood glucose level. Diabetes is commonly associated with an elevated level of reactive carbonyl species such as α-dicarbonyl methylglyoxal (MGO) in serum and urine [11, 33]. Accumulation of MGO is mainly a result of hyperglycemia and increased oxidation of fatty acid. While a low level of reactive carbonyl species is necessary for some physiological processes such as cell growth, immune defense, and differentiation, an excessive concentration of MGO is a hallmark of several pathological conditions associated with neurodegeneration and diabetes [28]. At its high level, MGO can react with several amino acids (including proline, arginine, lysine, and threonine) to form glycated adducts that compromise the function of the altered proteins. If blood glucose levels are not properly controlled, patients with diabetes can develop cardiovascular diseases, including diabetic cardiomyopathy. These cardiomyopathies are usually associated with diastolic dysfunction as well as ventricular arrhythmias [17, 25, 19]. Carbonylation of several key Ca^2+^ transport systems have been suggested contributing to [Ca^2+^]_i_ dysregulation during diabetic cardiomyopathy. It has been shown that carbonylation of SERCA2a and RyR2 reduces SR Ca^2+^ uptake and desynchronize SR Ca^2+^ release, causing diastolic dysfunction in the type 1 diabetic rat model [29, 30]. NKA is also a suitable substrate for carbonylation in renal proximal tubules and this post translational modification reduces NKA function [35]. Despite its clinical significance, the effect of reactive carbonyl species on cardiac [Ca^2+^]_i_ and [Na^+^]_i_ regulation are still not fully understood.

The aim of this study was to determine mechanisms by which MGO affects intracellular Ca^2+^ and Na^+^ homeostasis in ventricular myocytes. By measuring intracellular [Ca^2+^] and [Na^+^] dynamics and NKA activity, we found that MGO affects intracellular Ca^2+^ regulation by increasing [Na^+^]_i_, primarily due to activation of a tetrodotoxin (TTX)-sensitive Na^+^ inward pathway. This, in turn, reduces the NCX-mediated Ca^2+^ extrusion, causing SR Ca^2+^ overload and pro-arrhythmogenic Ca^2+^ waves.

## MATERIALS and METHODS

### Isolation of ventricular myocytes.

All animal experiments were carried out in accordance with the National Institutes of Health guide for the care and use of Laboratory animals [20]. Male and female C57Bl6/J mice, (11 of male and 8 of female animals, Jackson Laboratories) were housed according to approved IACUC guidelines. Mice aged between 2–3 months were anesthetized using Isoflurane (1%). Following thoracotomy, hearts were quickly excised, immersed in Ca^2+^ free buffer, mounted on a Langendorff apparatus, and retrogradely perfused with the zero Ca^2+^ Tyrode buffer (in mM: NaCl 140; KCl 4; MgCl2 1; glucose 10; Hepes 10; pH 7.4). (37°C) containing Liberase H (Roche), according to a procedure described previously [9, 14]. The left ventricle was excised from the digested heart, placed in stop buffer containing BSA 1 mg/mL, cut into several pieces (average size 1 mm) and gently triturated into single cells. Myocytes were pelleted by gravity and resuspended in the low-Ca^2+^ Tyrode buffer (in mM: NaCl 140; KCl 4; CaCl_2_ 0.1; MgCl_2_ 1; glucose 10; Hepes 10; pH 7.4). [Ca^2+^] was gradually adjusted to 1 mM. Isolated cardiomyocytes were stored at room temperature (20°C). All chemicals and reagents were purchased from Sigma-Aldrich (St Louis, USA).

### Measurement of [Ca ^2+^] _i_.

Changes in the cytosolic [Ca^2+^] ([Ca^2+^]_i_) were measured with laser scanning confocal microscopy (Radiance 2000 MP, Bio-Rad, UK) equipped with a ×40 oil-immersion objective lens (N.A.=1.3) as described previously [38, 39]. To record [Ca^2+^]_i_, we used the high-affinity Ca^2+^ indicator Fluo-4 (Molecular Probes/Invitrogen, Carlsbad, CA, USA). To load the cytosol with Fluo-4, ventricular myocytes were incubated at room temperature with 10 μM Fluo-4 AM for 15 min in Tyrode solution (in mM: NaCl 140; KCl 4; CaCl_2_ 1; MgCl_2_ 1; glucose 10; Hepes 10; pH 7.4), followed by a 20 min wash. Fluo-4 was excited with the 488 nm line of an argon laser and the emission signal collected at wavelengths above 515 nm. Changes in [Ca^2+^]_i_ were expressed as changes in F/F_0_, where F_0_ is the Fluo-4 signal at the resting condition.

Ca^2+^ spark measurements were conducted in permeabilized ventricular myocytes as described previously [38–40]. After the surface membrane permeabilization with saponin, Ca^2+^ sparks were studied in an internal solution composed of (in mM): K-aspartate 100; KCl 15; KH_2_PO_4_ 5; MgATP 5; EGTA 0.35; CaCl_2_ 0.1; MgCl_2_ 0.75; phosphocreatine 10; HEPES 10; Fluo-4 pentapotassium salt 0.04 mM; dextran (MW: 40,000) 8%, and pH 7.2 (KOH). Free [Ca^2+^] and [Mg^2+^] in this solution were 100 nM and 1 mM, respectively. Sparks were detected and analyzed using the SparkMaster algorithm [27].

### Measurement of [Na ^+^] _i_.

Changes in [Na^+^]_i_ were monitored using inverted fluorescence microscope (Nikon Ti2 Eclipse) equipped with air immersion 40x objective and Lumencore Spectra X excitation system. The fluorescent sodium indicator SBFI/AM (Molecular Probes/Invitrogen, Carlsbad, CA, USA) was used to trace the Na^+^-dependent fluorescent signal. To load the cytosol with SBFI, ventricular myocytes were incubated at room temperature with 20 μM SBFI/AM for 30 min in Tyrode solution, followed by a 20 min wash. The SBFI signal was detected using the stream acquisition tool in Metamorph software (Molecular Devices) with 100 ms excitation (excitation 325/25 nm, emission 530 nm). Emitted light was passed through a dichroic emission filter cube (DAPI/GFP/TagRFP Spectra X emission filter set) and detected with Photometrics Prime 95B 25 mm camera. Two-dimensional images were obtained at 15 s intervals. Changes of [Na^+^]_i_ are presented as background-subtracted normalized fluorescence (1−F/F_0_). The experiments were conducted in Tyrode solution. All 2-D images and line scan measurements for [Ca^2+^]_SR_ were analyzed with ImageJ software (NIH, USA).

### Preparation of purified NKA.

A purified sodium pump was prepared from the pig’s outer medulla as described previously [23]. The SDS-solubilized microsomal membranes were separated using differential ultracentrifugation and the final protein was aliquoted at a concentration of 1 mg/ml and stored at −80°C.

### Measurements of NKA-specific ATPase activity.

NKA ATPase activity was determined by the Baginsky method as described previously [3]. The method detects the presence of inorganic phosphate in the sample by measuring absorbance at 710 nm. Specifically, 2 μl of purified NKA was mixed with 48 μl ATPase buffer (in mM: Na-ATP 3.3, NaCl 192, MgCl_2_ 4, KCl 20; pH 7.2) and incubated for 6 minutes at room temperature. The inorganic phosphate was stained with 75 μl of staining solution (160 mM ascorbic acid, 3.7% (v/v) HCl, 3% (w/v) SDS, and 0.5% ammonium molybdate) and the reaction was stopped after 8 minutes by addition of 125 μl of stopping solution (0.9% (w/v) bismuth citrate, 0.9% (w/v) sodium citrate and 3.7% HCl). Immediately after stopping the reaction, the plate was read using the microplate reader Clario Star (BMG Labtech, Germany). The calibration line was determined using the set of KH_2_PO_4_ solutions with concentrations ranging between 0–37.5 nM. To estimate the NKA-specific ATPase activity, the signal in the presence of the NKA inhibitor ouabain (50 μM) was subtracted from the total ATPase activity.

### Statistics.

Data are presented as mean ± standard error of the mean (SEM) of n measurements. Statistical comparisons between groups were performed using Student’s t test for unpaired data sets. Differences were considered statistically significant at P < 0.05. Statistical analysis and graphical representation of averaged data was carried out using the OriginPro7.5 software (OriginLab, USA).

## RESULTS

### Effect of protein carbonylation on Ca^2+^ regulation in mouse ventricular myocytes

**Figure 1:**
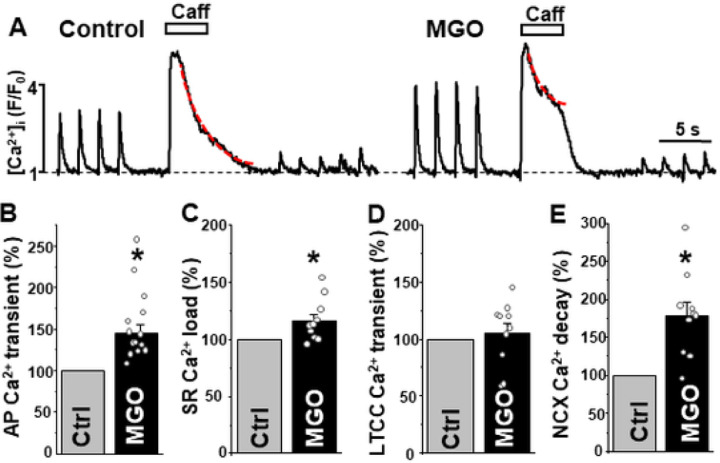
Effect of MGO on Ca^2+^ regulation in intact mouse ventricular myocytes. **A**, AP- and caffeine- induced Ca^2+^ transients in control conditions and during MGO (200 mM) application. The amplitude of Ca^2+^ transient during caffeine (10 mM) application was analyzed to assess changes in SR Ca^2+^ load. Duration of caffeine (Caff) applications is marked by white boxes. Effect of MGO on AP-induced Ca^2+^ transient amplitude **(B)**, SR Ca^2+^ load **(C)**, LTCC-mediated Ca^2+^ transient amplitude **(D)** and NCX-mediated [Ca^2+^]_i_ decay rate **(E)**. The data were collected from 12 myocytes isolated from 4 animals. *P<0.05 vs Control.

To determine how protein carbonylation affects cardiac Ca^2+^ signaling, we studied the effect of the reactive α-dicarbonyl methylglyoxal (MGO) on action potential (AP)- and caffeine- induced Ca^2+^ transients in mouse ventricular myocytes (Fig. 1A). Myocytes were electrically stimulated at 0.5 Hz to evoke AP-induced Ca^2+^ transients. The amplitude of Ca^2+^ transient during caffeine (10 mM) application was analyzed to estimate changes in SR Ca^2+^ load. MGO (200 μM) increased AP-induced Ca^2+^ transients to 145 ± 11% (n = 12 cells; Fig. 1B) and SR Ca^2+^ load to 116 ± 6% (n = 12 cells; Fig. 1C) compared to 100% in control. As a result, MGO enhanced the fractional release (Ca^2+^ transient/Ca^2+^ load) by 26%. The maximal effect of MGO on AP-induced Ca^2+^ transient developed within 3–5 min.

The first AP-induced Ca^2+^ transient after complete depletion of [Ca^2+^]_SR_ with caffeine is mainly mediated by Ca^2+^ influx via L-type Ca^2+^ channels (LTCC) [10]. Analysis of the LTCC-mediated Ca^2+^ transient amplitude suggested that MGO does not significantly affect LTCC activity (Fig. 1D). MGO drastically slowed down the rate of [Ca^2+^]_i_ decay during the caffeine application (Fig. 1E), but not during AP. As the Ca^2+^ decay during the caffeine application is predominantly mediated by NCX-mediated Ca^2+^ extrusion [4], these results suggest that carbonylation reduces cytosolic Ca^2+^ extrusion by NCX. Consequently, more cytosolic Ca^2+^ could be pumped by SERCA2a into the SR, leading to an increase in SR Ca^2+^ load.

**Figure 2:**
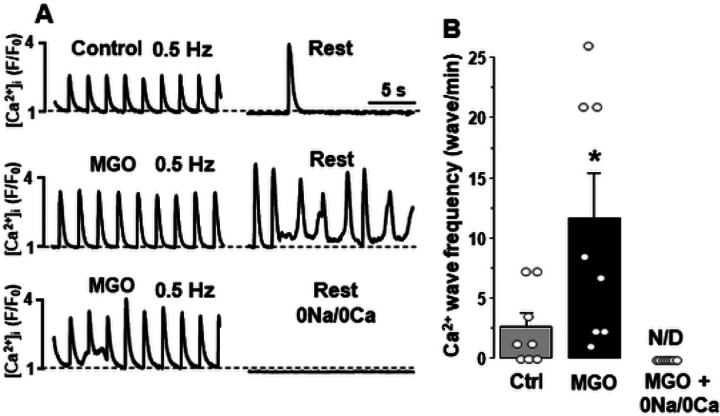
Effect of MGO on spontaneous Ca^2+^ waves during rest in intact ventricular myocytes. **A,** AP-induced Ca^2+^ transients (left panel) and spontaneous Ca^2+^ waves during rest (right panel) in control conditions, during MGO (200 mM) application and during MGO application in the presence of the 0 Ca^2+^/0 Na^+^ Tyrode solution. **B,** effect of MGO on Ca^2+^ wave frequency during rest. The data were collected from 8 myocytes isolated from 3 animals. *P<0.05 vs Control. N/D – not detected.

Next, we studied whether an increase in SR Ca^2+^ load caused by MGO increases the propensity of spontaneous SR Ca^2+^ release events. After 10 min electrical pacing (0.5 Hz) to stabilize SR Ca^2+^ load, Ca^2+^ waves were recorded during a following period of rest (Fig. 2A). MGO (200 μM) increased Ca^2+^ wave frequency from 2.7 ± 1.1 to 11.7 ± 3.7 waves/min (n = 8 cells) or more than 4 times (Fig. 2B). The Ca^2+^/Na^+^ free Tyrode solution abolished the spontaneous Ca^2+^ release events in the presence of MGO (Fig. 2B). These results suggest that the reduced Ca^2+^ extrusion by NCX and SR Ca^2+^ overload increase the propensity of spontaneous SR Ca^2+^ release events during protein carbonylation.

**Figure 3:**
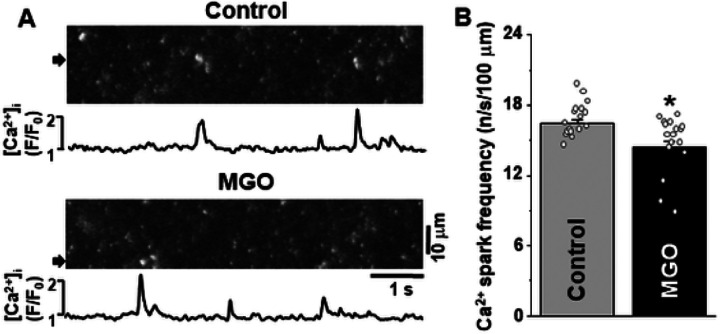
Effect of MGO on spontaneous Ca^2+^ sparks in permeabilized ventricular myocytes. **A,** confocal line scan images of [Ca^2+^]_i_ with corresponding F/F_0_ profiles of Ca^2+^ sparks in control conditions and during MGO (200 mM) application. F/F_0_ profiles were obtained by averaging the fluorescence over the 1 mm wide regions indicated by the black arrows. **B,** Effect of MGO on Ca^2+^ spark frequency. The data were collected from 17 myocytes isolated from 4 animals. *P<0.05 vs Control.

To determine whether MGO directly affects the RyR-mediated Ca^2+^ leak, we studied the effect of MGO on Ca^2+^ sparks in permeabilized ventricular myocytes (Fig. 3A). Studying permeabilized cells enabled exclusion of sarcolemmal Ca^2+^ transporters from SR Ca^2+^ regulation. In the control conditions, Ca^2+^ sparks were detected at a stable frequency of 16.5 ± 0.4 sparks s^−1^ (100 μm)^−1^. MGO (200 μM) decreased Ca^2+^ spark frequency to 14.4 ± 0.6 sparks⋅s^−1^ ⋅ (100 μm)^−1^ or by 13% (n = 17 cells; Fig. 3B). The inhibitory effect on Ca^2+^ spark frequency developed within 3 min and remained stable during MGO application (15 min). The inhibitory effect of MGO on Ca^2+^ spark frequency was associated with a decrease of SR Ca^2+^ load by 14 ± 3% (n = 10 cells).

### Effect of protein carbonylation on Ca^2+^ regulation during adrenergic receptor activation

**Figure 4:**
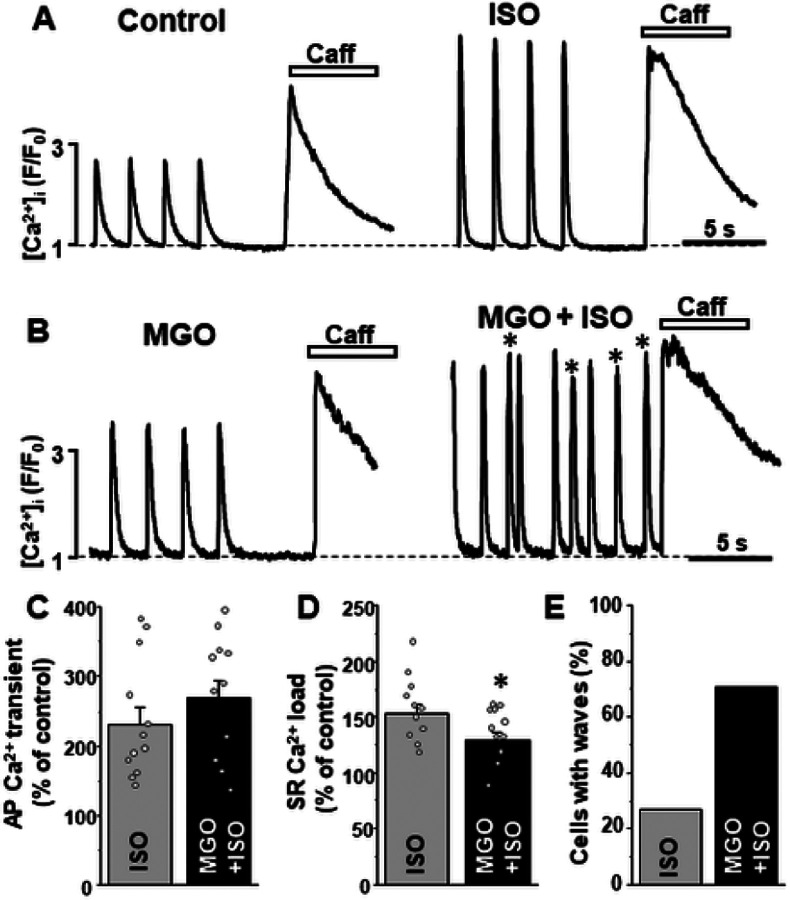
Effect of MGO on Ca^2+^ signaling during adrenergic receptor activation. **A,** F/F_0_ profiles of changes in [Ca^2+^]_i_ during AP- and caffeine- induced Ca^2+^ transients in control conditions, during adrenergic receptor activation with ISO (0.1 mM). **B,** F/F_0_ profiles of changes in AP- and caffeine- induced Ca^2+^ transients in the presence of MGO (200 mM) and during the consecutive application of ISO (0.1 mM). Ca^2+^ waves are marked with asterisk. Effect of ISO in control conditions and the presence of MGO on AP-induced Ca^2+^ transient amplitude **(C)**, SR Ca^2+^ load **(D)** and percentage of myocytes with spontaneous Ca^2+^ waves **(E)**. A total of 11 myocytes isolated from 3 animals were studied in these experiments. *P<0.05 vs ISO alone.

b-adrenergic receptor (b-AR) activation with isoproterenol (ISO; 0.1 μM) increased the AP-induced Ca^2+^ transient amplitude to 230 ± 25% (n = 11 cells) and SR Ca^2+^ load to 153 ± 9% compared to 100% in control (n = 12 cells; Figs. 4A, C and D). This effect is mainly mediated by an increase in SR Ca^2+^ uptake and sarcolemmal Ca^2+^ influx due to phospholamban and LTCC phosphorylation by protein kinase A (PKA) [6, 15]. We studied whether protein carbonylation affects the β-AR stimulation effects on Ca^2+^ signaling. These experiments revealed that in the presence of MGO (200 μM), ISO increased the AP-induced Ca^2+^ transient amplitude to 268 ± 27% (n = 11 cells; Fig. 4B) and SR Ca^2+^ load to 130 ± 7% (n = 11 cells; Fig. 4C) compared to 100% in control. The results between control and MGO groups were not statistically different, suggesting that the effect of adrenergic stimulation on Ca^2+^ regulation remained largely preserved during protein carbonylation. In ventricular myocytes, SR Ca^2+^ overload induced by adrenergic stress can lead to spontaneous SR Ca^2+^ release in the form of diastolic Ca^2+^ waves [7]. We found that in control conditions, 3 myocytes out of 11 studied or 27% exhibited diastolic Ca^2+^ waves during b-AR activation with ISO. Treatment of cells with MGO increased the fraction of myocytes with Ca^2+^ waves during adrenergic stimulation to 71% or more than 3 times (Fig. 4B and E).

### Effect of protein carbonylation on [Na^+^]_i_ and NKA-dependent ATPase activity

**Figure 5:**
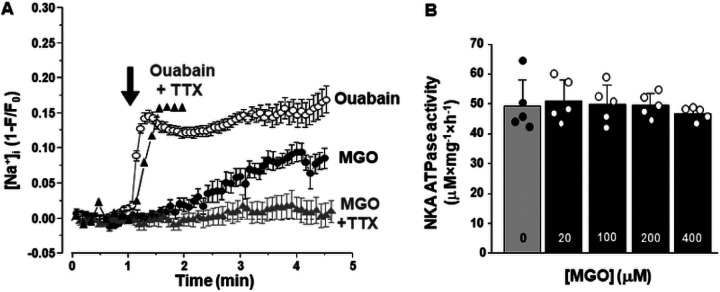
Effect of MGO and ouabain on intracellular [Na^+^] homeostasis and NKA-dependent ATPase activity. **A,** Effect of MGO (200 mM; black circles) and ouabain (20 mM; empty circles) on [Na^+^]_i_ in intact ventricular myocytes. Effect of MGO (gray triangles) and ouabain (black triangles) on [Na^+^]_i_ in the presence of TTX (10 mM). The black arrow marks the application either MGO or ouabain. [Na^+^]_i_ was measured with SBFI. 15 myocytes were studied to measure the effect of MGO, and 10 myocytes were studied to measure the effect of ouabain. Myocytes were isolated from 3 animals. **B,** Effect of MGO on NKA-dependent ATPase activity. The pump activity was measured as an accumulation of inorganic phosphate sensitive to the NKA inhibitor ouabain.

To assess whether the decreased NCX-mediated Ca^2+^ extrusion induced by MGO was a result of elevated intracellular [Na^+^] (Na^+^]_i_), we measured changes in [Na^+^]_i_ in intact ventricular myocytes. We found that MGO (200 μM) increased [Na^+^]_i_ by 57% from the maximal effect produced by the selective Na^+^-K^+^ ATPase (NKA) inhibitor ouabain (20 μM). In contrast to ouabain which caused the maximal [Na^+^]_i_ elevation within 1 min of its application, the effect of MGO on [Na^+^]_i_ required more than 3 min to fully develop (Fig. 5A). These results suggest that protein carbonylation reduces the NCX-mediated Ca^2+^ extrusion by increasing cytosolic [Na^+^].

Since NKA is the main mechanism that controls cytosolic [Na^+^], we tested whether carbonylation affects the NKA-dependent ATPase activity. NKA samples were preincubated with different MGO concentrations for 60 min at room temperature and the ouabain-specific ATPase activity was measured as accumulation of inorganic phosphate [3]. These experiments revealed that MGO does not affect the NKA-dependent ATPase activity (Fig. 5B). It has been shown that an accumulation of [Na^+^]_i_ in ventricular myocytes from failing heart was sensitive to the Na^+^ channel inhibitor TTX [13]. We found that TTX (10 μM) significantly prevented [Na^+^]_i_ accumulation induced by MGO. In the presence of TTX, MGO (200 μM) increased [Na^+^]_i_ only by 11% from the maximal effect produced by ouabain (20 μM). At the same time, TTX did not prevent an increase in [Na^+^]_i_ during the ouabain application (Fig. 5A).

### Effect of elevated [Na^+^]_i_ on Ca^2+^ regulation

**Figure 6:**
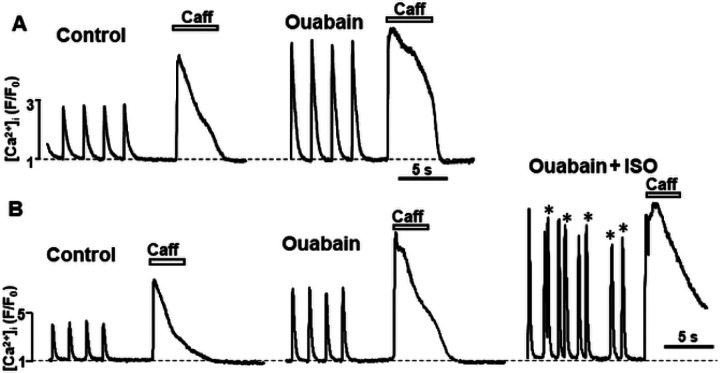
Effect of ouabain on Ca^2+^ signaling in control conditions and during adrenergic receptor activation. **A,** F/F_0_ profiles of changes in [Ca^2+^]_i_ during AP- and caffeine- induced Ca^2+^ transients in control conditions and during NKA inhibition with ouabain (20 mM). **B,** F/F_0_ profiles of changes in AP- and caffeine- induced Ca^2+^ transients in control conditions, the presence of ouabain (20 mM) and during the consecutive application of ISO (0.1 mM). Ca^2+^ waves are marked with asterisk. A total of 10 myocytes isolated from 2 animals were studied in these experiments.

If the elevated [Na^+^]_i_ is a main factor that causes SR Ca^2+^ overload during protein carbonylation, then an increase of [Na^+^]_i_ alone should produce a similar effect on Ca^2+^ regulation as MGO. To selectively increase [Na^+^]_i_, NKA pump was inhibited with ouabain. We found that ouabain (20 μM) increased an amplitude of AP-induced Ca^2+^ transients to 187 ± 10%, increased SR Ca^2+^ load to 147 ± 7% and slowed down NCX-mediated Ca^2+^ extrusion by 272 ± 27% (n = 12 cells; Fig. 6A). In the presence of ouabain, ISO (0.1 μM) increased the AP-induced Ca^2+^ transient amplitude to 223 ± 6% and increased SR Ca^2+^ load to 185 ± 8% (n = 10; Fig. 6B). Similar to the MGO effect, ouabain increased propensity of spontaneous Ca^2+^ waves in cells treated with ISO from 17 to 65% or more than 3 times.

## DISCUSSION

Cardiomyopathy is one of the leading causes of morbidity and mortality in type 1 and type 2 diabetic patients. Among several factors, increased production of reactive carbonyl species is viewed as a critical step involved in the development of diabetic cardiomyopathy [33]. It has been shown that myocardium from patients with diabetic heart failure (HF) exhibits the increased level of carbonylation of the sarcomeric proteins as compared with myocardium from non-diabetic HF patients [26]. Several studies have shown that in hearts from the type 1 diabetic animal model, RyR2 and SERCA2a are characterized by highly glycated MGO-adducts [29, 30]. These modifications have been proposed altering the function of these Ca^2+^ handling proteins and, therefore, intracellular Ca^2+^ dynamics. It has been shown that in myocytes from type 1 diabetic hearts RyR2-mediated Ca^2+^ sparks were significantly increased and AP-evoked Ca^2+^ transients were largely dyssynchronous [31]. Moreover, SERCA2a-mediated Ca^2+^ uptake decreased in type 1 diabetic hearts and this effect was associated with reduced the affnity of SERCA2a for Ca^2+^ [24]. Moreover, it has been shown that cardiomyocytes contraction and Ca^2+^ signaling were depressed in the type 2 diabetic rat model together with reduced RyR2 expression level [16]. The type 2 diabetics hearts were also characterized by reduced SERCA2a function [34]. While carbonylation of RyR2 and SERCA2a might contribute to Ca^2+^ mishandling in the diabetic heart, the effect of reactive carbonyl species on intracellular Ca^2+^ regulation is still not fully understood. Moreover, an alteration of Ca^2+^ homeostasis in the diabetic heart is likely to be a result of complex changes of many intracellular processes, including cell energetics, gene regulation, protein expression and protein post-translational modifications (e.g., oxidation, phosphorylation, and carboxylation). To define specific molecular mechanisms by which carboxylation affects cardiac Ca^2+^ regulation, we characterized the effect of the reactive carbonyl byproduct MGO on Ca^2+^ dynamics in mouse ventricular myocytes.

Using confocal microscopy and in-cell Ca^2+^ imaging, we found that MGO increases the amplitude of Ca^2+^ transients triggered by the action potential. The effect was accompanied by an increase in SR Ca^2+^ load (Fig. 1). MGO also enhanced the fractional SR Ca^2+^ release and increased propensity of spontaneous Ca^2+^ waves during rest (Fig. 2). These results are in good agreement with the previously published work [30] that suggests that RyR2 carboxylation increases RyR2 activity. In permeabilized myocytes, however, MGO reduced spontaneous Ca^2+^ spark frequency, and this effect was associated with a decrease in SR Ca^2+^ load (Fig. 3). Since Ca^2+^ spark frequency highly depends on SR Ca^2+^ load [36], the observed decrease spark activity can be explained by activation of RyR2-mediated Ca^2+^ leak by MGO with following a depletion of SR Ca^2+^ load. However, this mechanism alone cannot explain the observed differences in the MGO effect on SR Ca^2+^ load. While MGO increased SR Ca load in intact myocytes, it decreased SR Ca load in permeabilized cells. Such discrepancy suggests that other cellular mechanisms of the MGO action have been disrupted after the sarcolemma permeabilization. Analysis of Ca^2+^ signaling in intact myocytes revealed that MGO significantly slows down the decay of Ca^2+^ transient during caffeine application (Fig. 1). As this decay is predominantly mediated by NCX-mediated Ca^2+^ extrusion [4], these findings suggest that protein carbonylation reduces cytosolic Ca^2+^ removal by NCX. Subsequently, more cytosolic Ca^2+^ could be pumped into the SR by SERCA2a, leading to an increase in SR Ca^2+^ load. We hypothesized that MGO might reduce the NCX-mediated Ca^2+^ extrusion via a Na^+^-dependent mechanism, similar to the positive inotropic effect produced by glycosides. Similar to the MGO effect on Ca^2+^ regulation, we observed that an increase of [Na^+^]_i_ after treating cells with a the selective NKA inhibitor ouabain slows down the NCX-mediated Ca^2+^ extrusion, increases the AP-triggered Ca^2+^ transients, and increases SR Ca^2+^ load (Fig. 6). Direct measurements of [Na^+^]_i_ revealed that MGO significantly increased [Na^+^]_i_ (Fig. 5). Since NKA is the main mechanism that controls cytosolic [Na^+^], we tested whether protein carbonylation directly inhibits NKA. However, analysis of the ouabain-specific ATPase activity revealed that MGO does not affect NKA function (Fig. 5). It has been shown that an accumulation of [Na^+^]_i_ in ventricular myocytes from failing heart was sensitive to the Na^+^ channel inhibitor TTX [13]. Here, we tested whether the MGO effect on [Na^+^]_i_ was also sensitive to TTX. Measurements of [Na^+^]_i_ revealed that TTX significantly prevented the [Na^+^]_i_ increase induced by MGO (Fig. 5). Thus, this study, for the first time, uncovered a new mechanism by which reactive carbonyl species can affect [Ca^2+^]_i_ in ventricular myocytes. This mechanism involves [Na^+^]_i_ accumulation due to activation of Na^+^ influx via a TTX-sensitive pathway.

Intuitively, protein carbonylation could be viewed as beneficial post-translational modification for Ca^2+^ regulation and, therefore, for heart function. However, as the inward Na^+^ gradient plays a critical role in many cellular processes, persistent cytosolic Na^+^ overload would have a negative impact on cardiac function. First, [Na^+^]_i_ accumulation would reduce the efficacy not only Ca^2+^ transport by NCX, but regulation of intracellular pH by the Na^+^-H^+^ exchanger and the Na^+^-driven HCO_3_^−^ transport. It would also reduce the transport of glucose, pyruvate, and several amino acids, therefore, unbalancing cellular metabolism. Moreover, protein carboxylation with MGO significantly increases the propensity of spontaneous Ca^2+^ waves during adrenergic receptor activation (Fig. 4). It appears that SR Ca^2+^ overload due to the reduced NCX function and the increased RyR2 activity create an effective substrate for Ca^2+−^ dependent cardiac arrythmias during adrenergic stress. Additionally, these spontaneous Ca^2+^ waves would reduce the efficacy of myocardial relaxation, increasing ventricular stiffness during diastole. In fact, it has been reported that diabetic cardiomyopathies are commonly associated with ventricular arrhythmias as well as diastolic dysfunction [17, 19]. Therefore, a therapeutic approach aimed at limiting protein carbonylation could be beneficial for protecting cardiac function in diabetic patients.

## Data Availability

The data supporting this article and other findings are available within the manuscript, figures, and from the corresponding authors upon request.
